# 

**Published:** 1821-09

**Authors:** 


THE LONDON
### Medical and Physical Journal.

3 OF VOL. XLVI.] SEPTEMBER, 1821. [NO. 271.

### NOTICE.

*Another of the series of* PRÖEMIA *to the several volumes of this Journal, (which commenced with that to the forty-third volume,) comprising a History of the Progress of Medicine and its auxiliary Sciences for the half-year immediately previous to the period of their production, respectively, was published on the last day of July. One of the especial intentions of those* Pröemia, *is to present a comprehensive view of the state and progress of Medicine throughout Europe generally, and in the United States of America; an object that cannot be effected in the regular monthly Numbers of the Journal, because of the small extent of space which can be there appropriated to this purpose.*

### MONTHLY CATALOGUE OF MEDICAL BOOKS.

Remarks on the Epidemic Yellow-Fever, which has appeared at intervals on the south Coast of Spain since the year 1800. By Robert Jackson, M.D. 8vo. 7s.

A Familiar Treatise on Disorders of the Stomach and Bowels, Bilious and Nervous Affections; with an Attempt to correct the most prevalent Errors in Diet, Exercise, &c. Being an Exposition of the most approved Means for the Improvement and Preservation of Health. Also, a Refutation of the Arguments urged by Sir Richard Phillips against the use of Animal Food. Containing likewise the Author's Opinion of the most probable Consequences of many prevailing Habits in Society, with practical Hints for their Prevention. By George Shipman, Member of the Royal College of Surgeons in London.

A Treatise on Dyspepsia, or Indigestion; with Observations on Hypochondriasis and Hysteria. The second Edition, greatly enlarged. By J. Woodforde, M.D. 5s. boards.

Pharmacopœia Equina, or New Pharmacopœia for Horses. By Bracy Clark, F.L.S. &c. &c.

A Catalogue of Books on Anatomy, Medicine, Surgery, Midwifery, Materia Medica, Chemistry, Botany, &c. sold by Samuel Highley, Medical Bookseller, 174, Fleet-street. To which is added, a List of all the Lectures commencing in October next, with the Terms, Hours of Attendance, &c. &c.

